# Effects of Augmented Reality Interventions on the Function of Upper Extremity and Balance in Children With Spastic Hemiplegic Cerebral Palsy: A Randomized Clinical Trial

**DOI:** 10.3389/fneur.2022.895055

**Published:** 2022-06-21

**Authors:** Wardah Hussain Malick, Rizwana Butt, Waqar Ahmed Awan, Muhammad Ashfaq, Qamar Mahmood

**Affiliations:** ^1^Physiotherapy Department, National Institute of Rehabilitation Medicine, Islamabad, Pakistan; ^2^Faculty of Rehabilitation and Allied Health Sciences, Riphah College of Rehabilitation and Allied Health Sciences, Riphah International University, Islamabad, Pakistan

**Keywords:** augmented reality, balance, cerebral palsy, function, spasticity, upper extremity

## Abstract

**Objective:**

To determine the effects of augmented reality (AR) interventions on the function of the upper extremity (UE) and balance in children with spastic hemiplegic cerebral palsy (SHCP).

**Methods:**

In total, 30 children with SHCP, aged 6 to 12 years, were randomly divided into three interventional groups. Each group received an AR game, i.e., Balance It, Bubble Pop, or Scoop'd (WonderTree, Pakistan). The UE function and balance were assessed at the baseline and after 8 weeks of intervention through the Disability of Arm, Shoulder, and Hand (DASH) questionnaire and Pediatric Balance Scale (PBS), respectively. The mixed ANOVA was used to determine the combined with-in and between-the-groups differences in the function of the upper extremity. The Wilcoxon sign ranked test was used for with-in group changes, while the Kruskal Wallis test with the bonferroni correction *post-hoc* analysis was used to compare the groups in terms of balance. The data were analyzed by using SPSS version 21 and the level of significance was set at *p* < 0.05. Paired sample *t*-test and Wilcoxon signed-rank test was used for analyzing the changes in the total DASH and PBS scores within the groups, respectively. One-way ANOVA was used to determine the differences between the groups in the total DASH and PBS scores, while the Kruskal Wallis test was used for the differences between the groups in the PBS items. The data were analyzed by using SPSS version 21.

**Results:**

All the groups improved significantly in the total DASH and PBS scores post-intervention. A significant difference was determined in standing with one foot in front between Bubble Pop and Balance It groups (*p* = 0.03). The total score of PBS also showed a significant difference between Bubble Pop and Balance It groups (*p* = 0.02).

**Conclusion:**

The AR interventions used in this study were found to be effective in improving the UE function and balance of children with SHCP. The Balance It game showed more promising results in improving the balance as compared with the other games, however, no significant difference was determined between the three AR games in terms of the UE function of the participants.

## Introduction

Cerebral palsy (CP) is a group of permanent disorders of movement and posture causing activity limitations which are attributed to non-progressive disturbances in the brain occurring early in the development (1). Associated conditions such as epilepsy, intellectual disability, and impairments of speech, hearing, or vision are common (2). The worldwide prevalence of CP is ~2.1 per 1,000 live births (3) and is the most common physical disability in children (4).

Spastic hemiplegic cerebral palsy (SHCP) is associated with motor impairments (contralateral to the involved brain hemisphere), including deficits in motor coordination and muscular weakness (5). This has a significant impact on the function of the upper extremity (UE) and can severely limit a child's ability to perform functional activities such as reaching, grasping, manipulating objects, or performing the activities of daily life (ADL), e.g., eating, dressing, and bathing (5). Due to asymmetric alignment, children with SHCP commonly bear weight through the unaffected lower extremity (LE) which, when combined with inherent spasticity, may cause muscle weakness and atrophy on the paretic side, growth retardation, and reduced balance (6). Poor postural control mechanism causes impairment of functional balance in children with CP which increases the risk of falls, thereby, affecting these children in the performance of ADLs, mobility, and participation (7).

Conventional approaches for the management of SHCP include medical, surgical, and rehabilitative strategies. Rehabilitation options typically include physiotherapy and occupational therapy (5). The use of interactive technology such as virtual reality (VR) and augmented reality (AR) in the rehabilitation of children with CP has been worth noticing (8, 9). VR embraces a completely immersive experience, while AR promotes the interaction between user, real-world, and digital content, therefore, displaying virtual images while remaining see-through capability (10). Hence, the AR environment provides a better sense of presence and reality judgments and thus has the advantage over VR in rehabilitation (11).

In AR, the user has a real-time visual perception of every movement of the body, which is based upon the concept of mirror therapy (12). Since AR facilitates natural body movements, the users of the AR system are more confident in making body movements than VR users (12), and the feedback through AR has been reported to increase normal activity during the motor relearning process (13). AR technology is an effective platform for performing a high number of repetitions of an activity, which, along with sensory input is required by the brain to acquire a skill (13, 14). In traditional neurological rehabilitation approaches, however, this becomes very challenging for the patients, and so, AR is a better option in this regard (14). AR games also engage the cognitive aspect of the patients, which leads to enhanced neuroplasticity (8). Furthermore, the fun element of these games is a major contributing factor in the meaningful engagement of the patient in the therapy session and adherence to the therapy program (15).

Augmented reality therapy is an emerging intervention for the rehabilitation of neurological conditions such as CP. Based on the literature, the effects of AR interventions on the function of UE and balance of children with SHCP are not well known. In the light of limited evidence to support AR games for children with CP, and the need to determine the impact on specific sub-groups, this study aimed to determine the effect of AR intervention on the function of UE and balance in children with SHCP and to compare different AR games for their effectiveness in improving these aspects.

## Methodology

### Study Design

A randomized clinical trial (registered with clinicaltrials.gov, NCT04171232) was conducted at the National Institute of Rehabilitation Medicine (NIRM), Islamabad, Pakistan, for a time duration of 5 months, i.e., from November 2019 to March 2020. The study was initiated post approval from the Institutional Review Board and Ethics Committee of NIRM (IRB serial number: NIRM-111/Physio/05). Written informed consent was taken from the participants' legal guardians/next of kin.

### Participants

A total of 45 children with SHCP were assessed for eligibility, who visited NIRM during the recruitment period. However, 30 children fulfilled the inclusion criteria and showed a willingness to participate in the study and were thus recruited through a non-probability convenient sampling technique. The treatment groups were named according to the game that each group played. The AR games used in this study were designed and developed by WonderTree, Pakistan.

The inclusion criteria were children with SHCP, aged between 6 and 12 years (16), who were independent and ambulant (classified as levels 1 or 2 on Gross Motor Functional Classification System), having a score of 1 or 2 on the Modified Ashworth Scale (in shoulder flexors, extensors, abductors, adductors, internal and external rotators, elbow flexors, extensors, forearm supinators, pronators, wrist flexors, and extensors), having good general health without any other neurological or orthopedic diagnoses as confirmed by health professionals and their parents, with sufficient cognitive capacity to understand the basic instructions and to cooperate with the physiotherapist during the assessment and the intervention.

Children with attention deficits or short attention span, having musculoskeletal abnormalities and contractures, who had received or planned to receive motor therapy such as botulinum toxin or constraint-induced movement therapy in the past 6 months or during the study, those with visual and hearing impairment or a history of seizures which could be provoked by TV lights (confirmed by the pediatric neurologist and physiotherapist) were excluded from the study.

### Randomization

**Figure 1:**
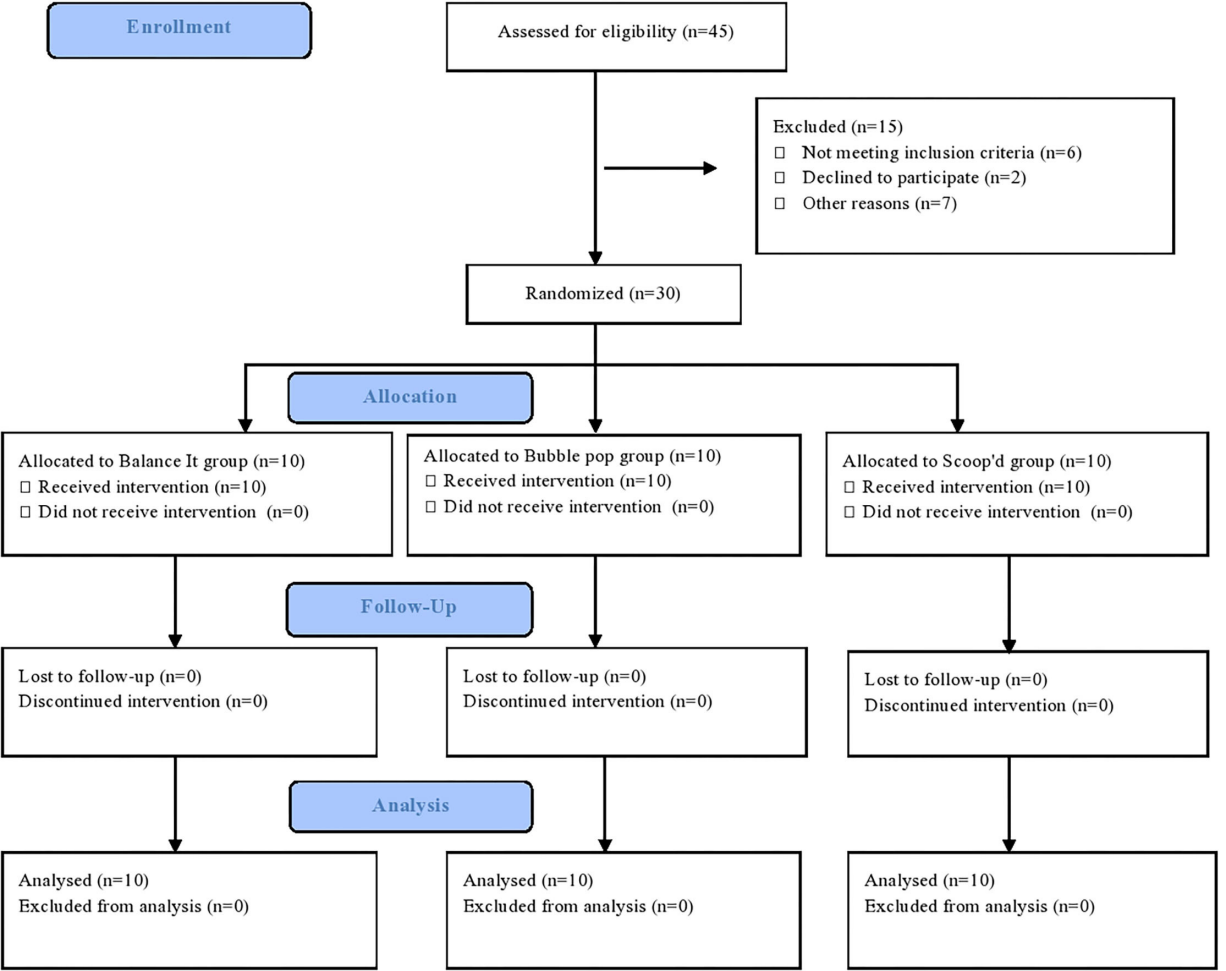
CONSORT diagram.

The participants were randomly divided into three treatment groups, i.e., Bubble Pop (*n* = 10), Scoop'd (*n* = 10), and Balance It (*n* = 10) (Figure 1). Randomization of the participants was done through the sealed envelope method by using a computerized random number generator. The sequence of random allocation was done by an individual who was not directly involved in the study. Consecutive random numbers were written on index cards and placed in thick and opaque sealed envelopes before the study. After taking consent from the participants' legal guardian/next of kin, the treating physiotherapist opened the envelope and gave the respective treatment to the patient. The study was single-blinded as assessing physiotherapist was blinded to the participants' intervention.

### Apparatus

**Figure 2:**
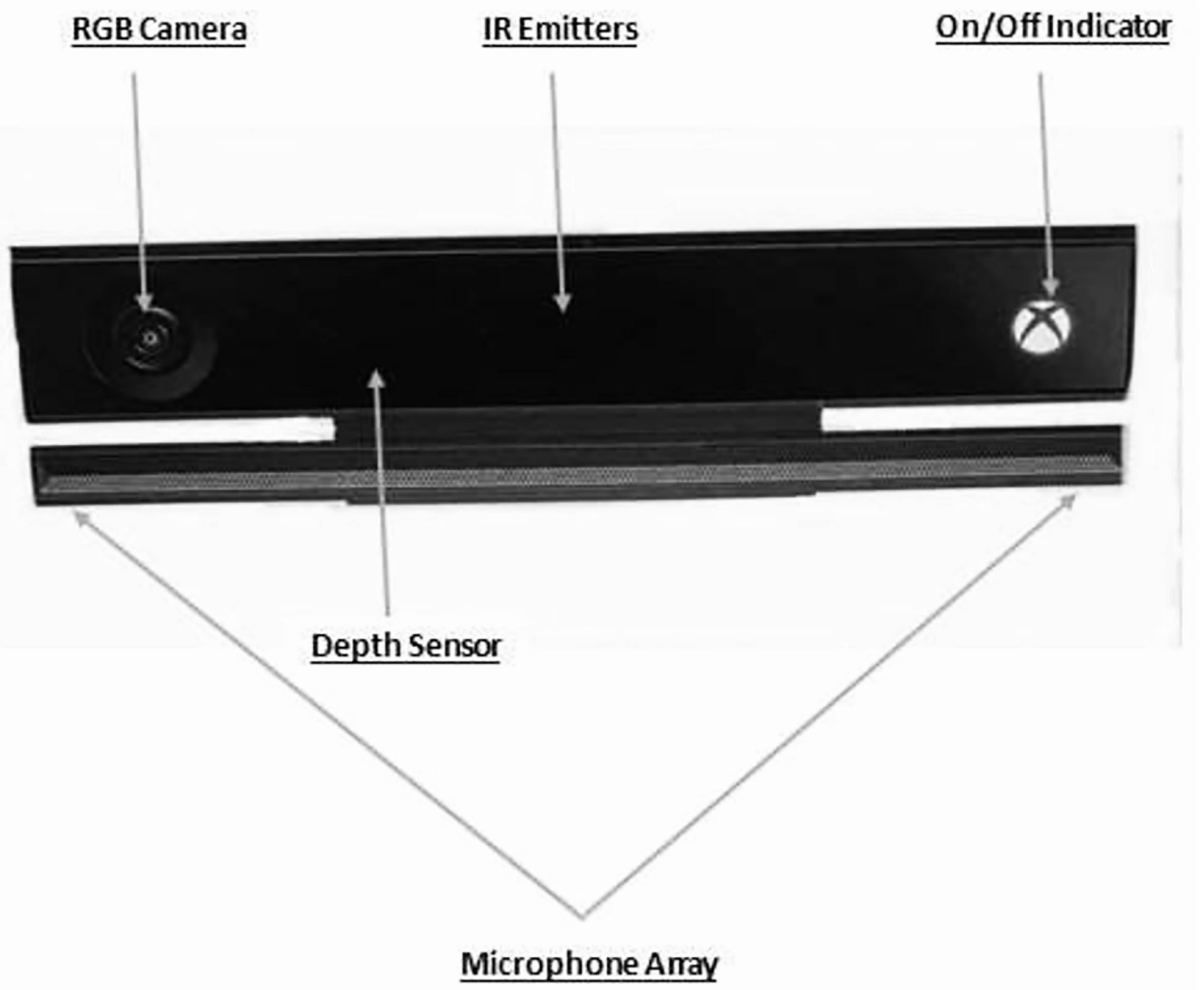
Microsoft Kinect v2 sensor.

The apparatus included a Microsoft Kinect v2 sensor (Figure 2), which consisted of an RGB camera, and three-dimensional sensors which detected the body and captured 3-D motion. Attached at the base of the Kinect was a mechanical drive that was used for tilting the sensor up and down (according to the height of the patient). The Kinect was attached to the PC through an adapter and took in the data of the user's joints and sent it to the PC (Core i5, 8 GB Ram, Windows 10 OS). The games were installed on the system and the Kinect was connected to the LED screen where the patient could see himself. Since the games involved the interaction between the user, digital content, and the real-world, and had the concept of mirror therapy incorporated, they were referred to as AR games. The system captured real-time movements through in-game processing and provided real-time visual feedback that encouraged the participants to avoid any compensatory movement.

### Intervention

All the participants received 24 sessions (three sessions in a week), for a time duration of 8 weeks at the National Institute of Rehabilitation Medicine (NIRM), Islamabad, Pakistan. Each session lasted for 15 min. Before the intervention, the AR games were explained to the children, and a five-minute-long trial session was given to all the participants for better understanding. The intervention sessions were conducted in a quiet room. The participants were instructed to stand 4 feet away from the projection screen on which they could see themselves in real-time. One participant played the AR game at a time and every session was supervised by the treating physiotherapist.

The participants were given verbal cues during the sessions to perform the required task and were encouraged to actively use both of their upper extremities. In each new session, the participants started with the game level which they had left in the last session. Furthermore, the total levels of the games were different, so an attempt was made to avoid any such influence on the results by providing the intervention for the same time duration to each group.

#### Bubble Pop

**Figure 3:**
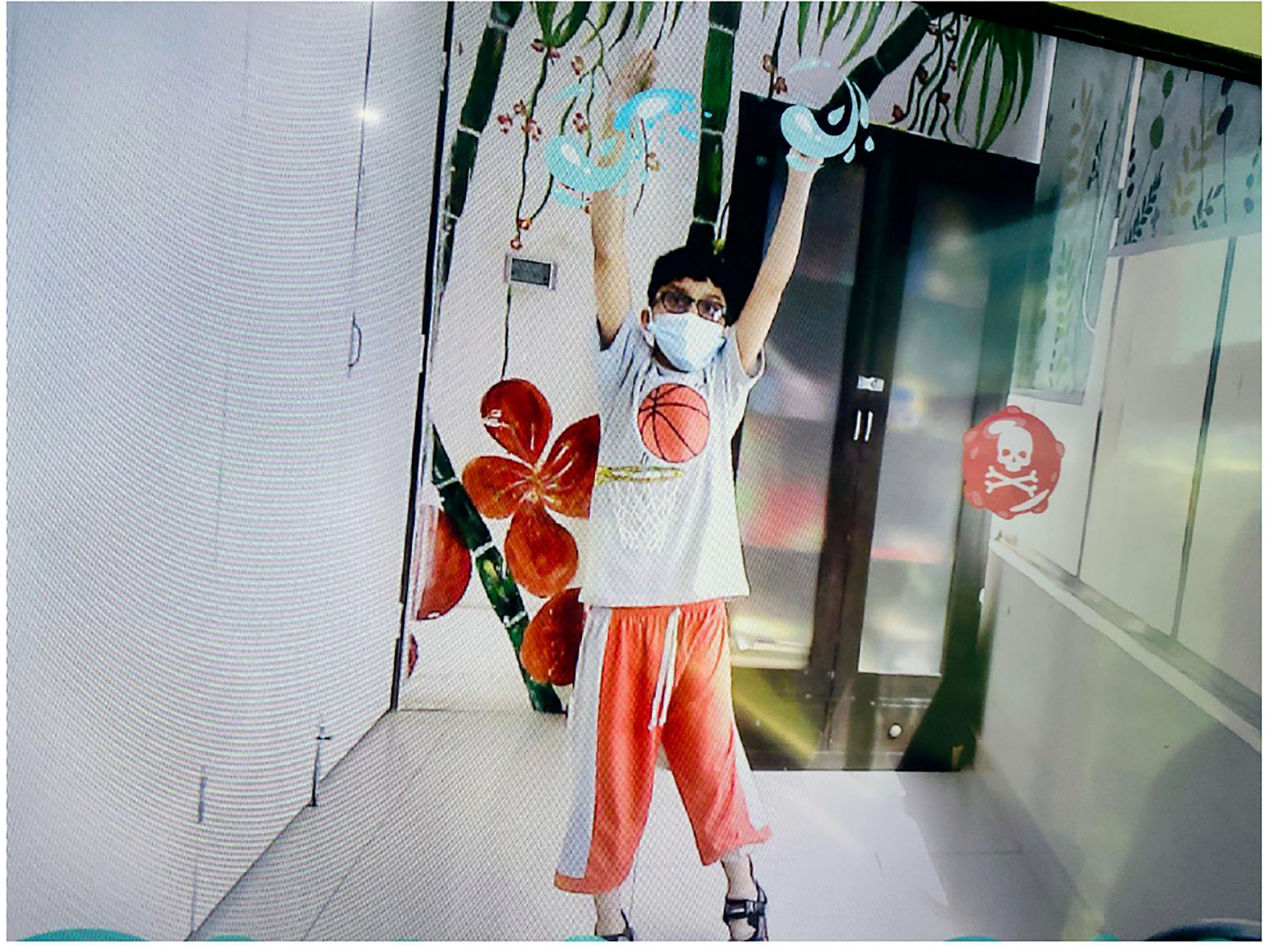
Bubble Pop (AR Game).

This AR-based therapeutic game (Figure 3) had 30 levels. The participants had to reach for the differently colored bubbles that appeared on the screen, coming from the random directions. They were instructed to pop the bubbles using their upper extremities and to avoid the red-colored bubbles which could negatively affect their game score. The participants were also suggested to move their whole body if required, to pop the bubbles on the screen. With each new level, the speed and number of bubbles increased which became increasingly challenging for the patients.

#### Scoop'd

**Figure 4:**
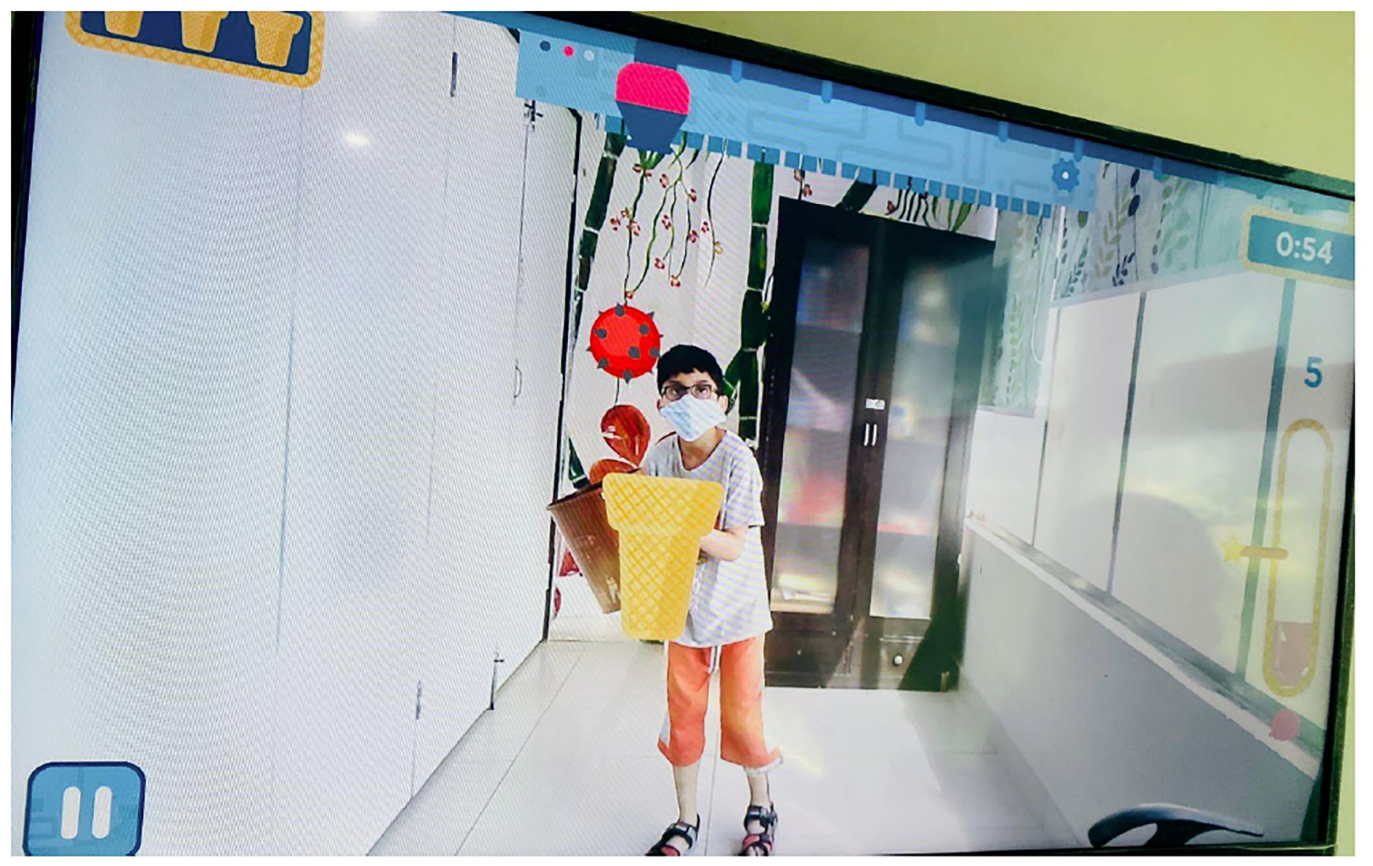
Scoop'd (AR Game).

This game (Figure 4) also consisted of 30 levels. The participant had to hold a basket (which resembled the ice-cream cone appearing on the screen) with both hands and had to catch the ice-cream scoops in it that fell at different spots. The participants were instructed to complete a specific game level at a particular time to reach the next level and were also advised to avoid the red-colored object which resulted in the deduction of their game score. The participants were also instructed to move their whole body if required, to catch the scoops. With each new level, the speed of the falling scoops increased, the time interval between two scoops decreased, and the color of the scoops also lightened. At the fifteenth level and onward, 1 kg weight was also added to the basket.

#### Balance It

**Figure 5:**
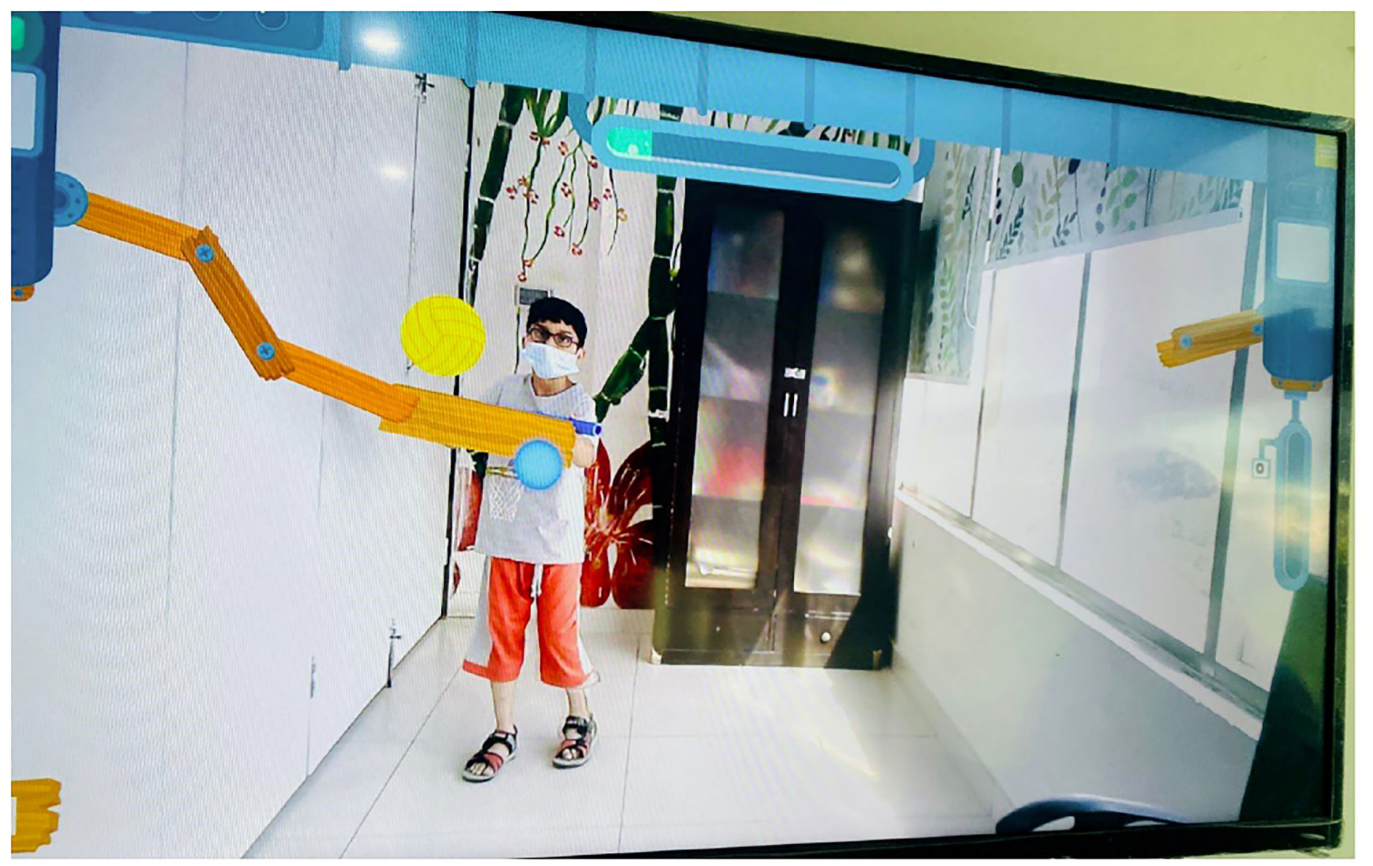
Balance It (AR Game).

Balance It (Figure 5) consisted of 15 levels. The participants were asked to raise and open both arms wide apart, in a way that they could hold an imaginary plank in their hands that appeared on the screen. During the first five levels of the game, the participants had to balance different objects on a plank (which fell from above) for a specific time duration. In the next five levels, the participant had to perform the same task as the previous levels, except that now they also had to move toward the opposite side and transfer the object from the plank to a basket in the corner. Furthermore, in the last five levels, the participants were instructed to balance two objects simultaneously on the plank and then transfer them to the basket, just like in the previous five levels of the game.

### Outcome Measures

Pediatric Balance Scale (PBS), which has established validity (17) and reliability (18), was used to assess the balance of the participants. Previous literature also supports the use of PBS to assess the functional balance of children with CP (19). Disability of Arm, Shoulder, and Hand (DASH) questionnaire (20) was used to assess the function of the impaired upper extremity, which has constructed validity and reliability (21). DASH has been used for the neurological conditions including CP previously as well (22). The data were collected pre-and post-intervention by a physiotherapist (23, 24). There were no participant dropouts during the eight-week interventional period. The researchers tried to ensure this by providing a transport facility for the participants and their parents.

### Statistics

The total score of DASH met the assumption of the parametric test so the mixed ANOVA was used to determine the combined with-in and between-the-groups differences in the function of the upper extremity. The total PBS score was not distributed normally, and the responses of PBS were on an ordinal scale, so the non-parametric Wilcoxon sign ranked test was used for with-in group changes, while the Kruskal Wallis test with the bonferroni correction *post-hoc* analysis was used to compare the groups. The effect size (partial eta squared Epsilon test, correlation coefficient, and Cohen's d) was also calculated, and the level of significance was set at *p* < 0.05. The SPSS version 21 was used for the statistical analysis.

## Results

In total, 30 children with SHCP participated in this study, of which 18 were males and 12 were females. The mean age of the participants was 8.76 ± 2.32 years. A comparison of the demographic and baseline characteristics revealed that there were no statistically significant differences among the three study groups with respect to the age, gender, side of impairment, GMFCS levels, and MAS grades at the start of the study. Therefore, all the groups were similar, comparable and homogenous in nature at the onset of the study (Table 1).

**Table 1:** Demographic and baseline characteristics of the study population.

**Demographic characteristics**	**Bubble Pop Group** **(*n* = 10)**	**Scoop'd Group** **(*n* = 10)**	**Balance It Group** **(*n* = 10)**	***P*-Value**
**Age (in years)**	8.5 ± 2.84	10 ± 2.11	7.8 ± 1.48	0.094^a^
**Gender**				
Male	6 (60%)	5 (50%)	7 (70%)	0.833^b^
Female	4 (40%)	5 (50%)	3 (30%)	
**Side of impairment**				
Left Side	4 (40%)	3 (30%)	8 (80%)	0.060^b^
Right side	6 (60%)	7 (70%)	2 (20%)	
**GMFCS level**				
Level 1	7 (70%)	5 (50%)	9 (90%)	0.148^b^
Level 2	3 (30%)	5 (50%)	1 (10%)	
**MAS score**				
Grade 1	6 (60%)	7 (70%)	5(50%)	0.659^b^
Grade 2	4 (40%)	3 (30%)	5 (50%)	

*The mean age with SD in each group is given*.

*^a^Parametric test was used to calculate p value*.

*^b^Non-parametric test was used to calculate p value*.

Within groups analysis (pre- and post-intervention) showed that the balance of the participants, assessed through PBS as the total score, improved significantly (*p* < 0.05) in all the groups (Table 2). The Scoop'd group improved significantly in standing with one foot in front (*p* = 0.03, *r* = −0.70) standing on one foot (*p* = 0.02, *r*= −0.67), and placing alternate foot on stool (*p* = 0.01, *r*= −0.77) components of PBS. Pre–post analysis of the Bubble-Pop group showed that standing with one foot in front (*p* = 0.03, *r*= −0.73), placing alternate foot on the stool (*p* = 0.01, *r* = −0.80), and reaching forward with an outstretched arm (*p* = 0.04, *r* = −0.65) improved significantly. A significant improvement was also determined in the Balance It group in standing with one foot in front (*p* = 0.03, *r* = −0.73).

**Table 2:** Pre-post analysis of PBS (with-in-groups).

**PBS items**		**Scoop'd**					**Bubble Pop**					**Balance It**				
		**Median**	**IQR**	**Z**	***p-value***	***R***	**Median**	**IQR**	**Z**	***p-value***	***R***	**Median**	**IQR**	**Z**	***p-value***	***R***
Sit to stand	Pre	4.00	0.00	−1.0	0.32	−0.31	4.00	0.25	0.0	1.00	0.00	4.00	0.00	0.0	1.00	0.00
	Post	4.00	0.00				4.00	0.25				4.00	0.00			
Stand to sit	Pre	4.00	0.00	0.0	1.00	0.00	4.00	0.00	0.0	1.00	0.00	4.00	0.00	0.0	1.00	0.00
	Post	4.00	0.00				4.00	0.00				4.00	0.00			
Transfers	Pre	4.00	0.25	−1.0	0.32	−0.31	4.00	1.00	−1.1	0.16	−0.44	4.00	0.00	−1.0	0.32	−0.31
	Post	4.00	0.00				4.00	1.00				4.00	0.00			
Stand unsupported	Pre	4.00	0.00	−1.0	0.32	−0.31	4.00	0.00	0.0	1.00	0.00	4.00	0.00	0.0	1.00	0.00
	Post	4.00	0.00				4.00	0.00				4.00	0.00			
Sit unsupported	Pre	4.00	0.00	−1.0	0.32	−0.31	4.00	0.00	0.0	1.00	0.00	4.00	0.00	0.0	1.00	0.00
	Post	4.00	0.00				4.00	0.00				4.00	0.00			
Stand with eyes closed	Pre	4.00	0.00	−1.0	0.32	−0.31	4.00	0.25	−1.0	0.32	−0.31	4.00	0.00	−1.0	0.32	−0.31
	Post	4.00	0.00				4.00	0.00				4.00	0.00			
Stand with feet together	Pre	4.00	0.25	−1.1	0.16	−0.44	4.00	0.25	−1.0	0.32	−0.31	4.00	0.25	−1.1	0.16	−0.44
	Post	4.00	0.00				4.00	0.25				4.00	0.00			
Stand with one foot in front	Pre	3.00	1.50	−2.3	**0.03**	−0.70	1.00	3.00	−2.3	**0.03**	−0.73	3.00	2.25	−2.3	**0.03**	−0.73
	Post	4.00	1.00				2.50	2.25				4.00	1.25			
Standing on one foot	Pre	2.00	2.25	−2.6	**0.02**	−0.67	1.00	3.00	−2.6	0.06	−0.43	3.00	3.00	−1.4	0.18	−0.65
	Post	3.00	1.25				2.50	2.00				3.00	2.00			
Turn 360 degrees	Pre	3.00	2.00	−1.0	0.06	−0.59	3.50	2.00	−1.9	0.06	−0.59	4.00	1.00	−1.3	0.08	−0.54
	Post	4.00	1.00				4.00	1.00				4.00	0.00			
Turn to look behind	Pre	4.00	1.25	−1.3	0.10	−0.51	4.00	1.25	−1.9	0.06	−0.59	4.00	0.00	−1.0	0.32	−0.31
	Post	4.00	0.00				4.00	0.00				4.00	0.00			
Retrieve object from floor	Pre	3.50	1.50	−1.1	0.13	−0.47	4.00	1.50	−1.4	0.18	−0.42	4.00	0.00	−1.0	0.32	−0.31
	Post	4.00	0.25				4.00	0.25				4.00	0.00			
Place alternate foot on Stool	Pre	3.00	2.25	−2.6	**0.01**	−0.77	2.50	2.50	−2.3	**0.01**	−0.80	3.50	1.00	−1.0	0.32	−0.31
	Post	4.00	1.00				3.50	1.00				4.00	1.00			
Reaching forward with Outstretched arm	Pre	3.50	1.25	−1.6	0.06	−0.58	3.50	4.00	−2.7	**0.04**	−0.65	4.00	1.00	−1.3	0.10	−0.51
	Post	4.00	0.00				4.00	1.00				4.00	0.00			
Total pediatric balance Scale score	Pre	47.50	6.75	−2.67	**0.01**	−0.84	45.00	16.75	−2.67	**0.01**	−0.84	52.50	6.00	−2.37	**0.02**	−0.74
	Post	53.50	2.75				51.00	8.50				54.50	3.75			

*PBS, Pediatric Balance Scale; IQR, inter-quartile ranges; level of statistical significance: p < 0.05. The bold values indicates statistically significant*.

**Figure 6:**
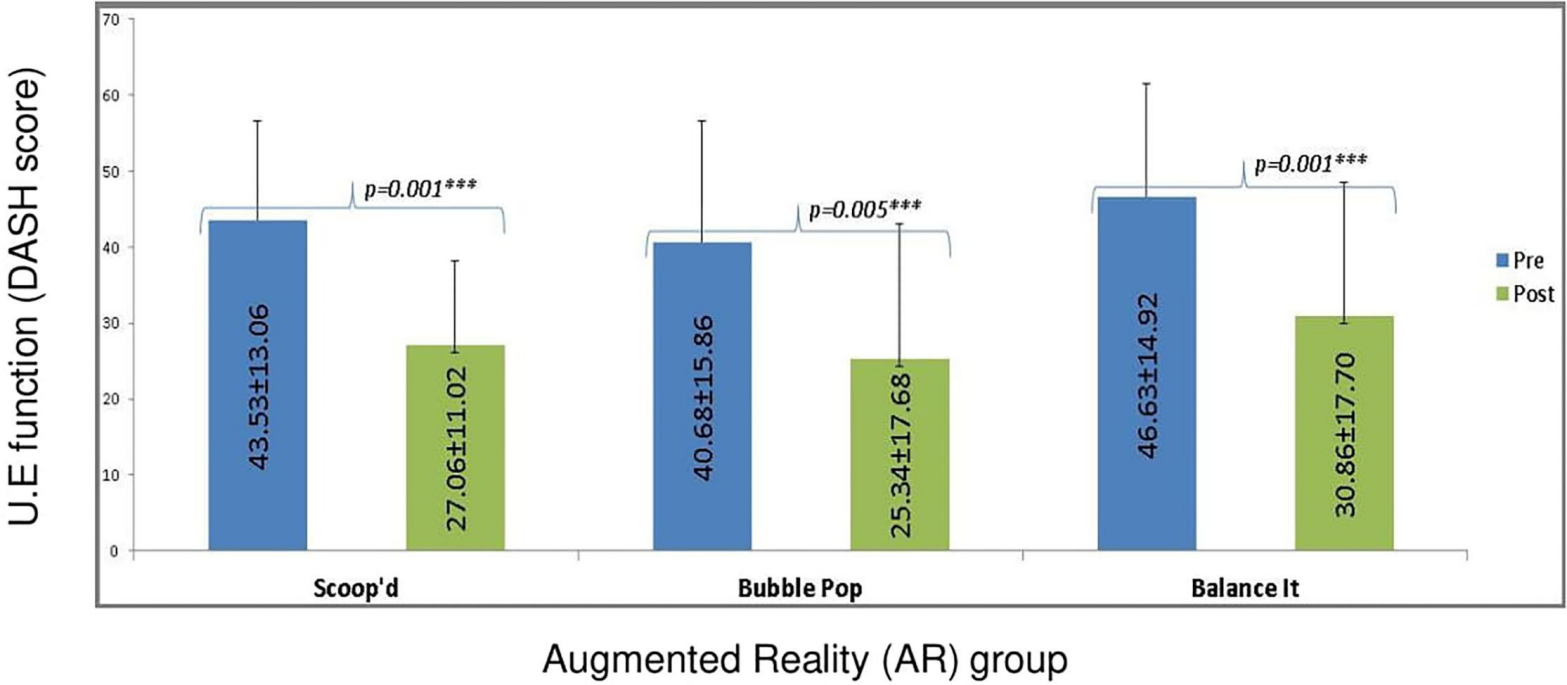
Pre- and Post-intervention analysis of DASH (with-in-groups). The *** symbol indicates the values which are statistically significant.

Similarly, a significant improvement was determined in the DASH score in all the groups, including Scoop d (*p* = 0.001, Cohen's *d* = 1.034), Bubble Pop (*p* = 0.005, Cohen's *d* = 1.037), and Balance It (*p* = 0.001, Cohen's *d* = 1.9) throughout the intervention (Figure 6). However, the result of mixed ANOVA showed no statistically significant interaction effects between the games and the time factor [F = 0.02(1, 27), *p* = 0.97]. However, the combined main effect showed statistically significant improvement [*F* = 61.61(1, 27), *p* < 0.001, η*p*^2^ = 0.69] with a large effect size in the DASH score.

The pre- and post-analysis between the interventional groups showed no statistically significant difference in the total DASH score [*F* = 0.41(2, 27), *p* = 0.67 η*p*^2^ = 0.03]. Moreover, the Kruskal–Wallis test showed that there was no statistically significant difference in the domains of PBS (*p* ≥ 0.05), except in standing with one foot in front [X^2^(2) = 7.401, *p* = 0.03, ε^2^ = 0.21] and in the total PBS score [X^2^(2) = 6.124, *p* = 0.04, ε^2^ = 0.25] with a large effect size post-intervention (Table 3). Furthermore, the *post hoc* analysis with bonferroni correction showed that there was a significant difference between the Bubble Pop and Balance It group in standing with one foot in front score [2.5(2.25) vs. 4(1.25), *p* = 0.03, *r* = 0.65]. The total score of PBS also showed a significant difference between Bubble Pop and Balance It groups [51(8.5) vs. 54.5(3.75), *p* = 0.02, *r* = 1]. However, there was no significant difference (*p* ≥ 0.05) between the Balance It and Scoop'd groups as well as between the Bubble Pop and Scoop'd group in standing with one front in front component and the total PBS score post-intervention.

**Table 3:** Between the groups' analysis of PBS (Pre- and Post-intervention).

**PBS items**		**Scoop'd**		**Bubble Pop**		**Balance It**		***X* ^2^**	***P*-value**
		**Median**	**IQR**	**Median**	**IQR**	**Median**	**IQR**		
Sit to stand	Pre	4.00	0.00	4.00	0.25	4.00	0.00	2.15	0.34
	Post	4.00	0.00	4.00	0.25	4.00	0.00	4.14	0.13
Stand to sit	Pre	4.00	0.00	4.00	0.00	4.00	0.00	0.00	1.00
	Post	4.00	0.00	4.00	0.00	4.00	0.00	0.00	1.00
Transfers	Pre	4.00	0.25	4.00	1.00	4.00	0.00	2.76	0.25
	Post	4.00	0.00	4.00	1.00	4.00	0.00	3.90	0.14
Stand unsupported	Pre	4.00	0.00	4.00	0.00	4.00	0.00	2.00	0.37
	Post	4.00	0.00	4.00	0.00	4.00	0.00	0.00	1.00
Sit unsupported	Pre	4.00	0.00	4.00	0.00	4.00	0.00	2.00	0.37
	Post	4.00	0.00	4.00	0.00	4.00	0.00	0.00	1.00
Stand with eyes closed	Pre	4.00	0.00	4.00	0.25	4.00	0.00	0.56	0.76
	Post	4.00	0.00	4.00	0.00	4.00	0.00	2.00	0.37
Stand with feet together	Pre	4.00	0.25	4.00	0.25	4.00	0.25	0.01	0.99
	Post	4.00	0.00	4.00	0.25	4.00	0.00	4.14	0.13
Stand with one foot in front	Pre	3.00	1.50	1.00	3.00	3.00	2.25	4.74	0.09
	Post	4.00	1.00	2.00	2.25	4.00	1.25	7.40	**0.03**
Standing on one foot	Pre	2.00	2.25	1.00	3.00	3.00	3.00	3.37	0.19
	Post	3.00	1.25	2.00	2.00	3.00	2.00	3.43	0.18
Turn 360 degrees	Pre	3.00	2.00	3.00	2.00	4.00	1.00	2.02	0.37
	Post	4.00	1.00	4.00	1.00	4.00	0.00	1.52	0.37
Turn to look behind	Pre	4.00	1.25	4.00	1.25	4.00	0.00	2.09	0.35
	Post	4.00	0.00	4.00	0.00	4.00	0.00	2.00	0.37
Retrieve object from floor	Pre	3.00	1.50	4.00	1.50	4.00	0.00	3.82	0.15
	Post	4.00	0.25	4.00	0.25	4.00	0.00	2.22	0.33
Place alternate foot on stool	Pre	3.00	2.25	2.00	2.50	3.00	1.00	3.06	0.22
	Post	4.00	1.00	3.00	1.00	4.00	1.00	0.50	0.78
Reaching forward with outstretched Arm	Pre	3.00	1.25	3.00	4.00	4.00	1.00	0.80	0.67
	Post	4.00	0.00	4.00	1.00	4.00	0.00	1.86	0.40
Total pediatric balance scale score	Pre	47.50	6.75	45.00	16.75	52.50	6.00	4.14	0.11
	Post	53.50	2.75	51.00	8.50	54.50	3.75	6.12	**0.04**

*PBS, Pediatric Balance Scale; IQR, inter-quartile ranges; level of statistical significance: p < 0.05. The bold values indicates statistically significant*.

## Discussion

The purpose of this study was to determine the effects of AR interventions on the function of UE and balance in children with SHCP and to compare the effects of different AR games on these aspects. According to the results of this study, after the completion of an 8-week training program with the AR interventions, the total score of the Pediatric Balance Scale (PBS) improved significantly in all the groups. Though lower-extremity exercises using AR have shown improved balance in various medical conditions previously as well (25), however, there is limited evidence available on the benefits of AR for balance training of individuals with cerebral palsy. Interactive computer play (ICP) is a modality in which the participant must actively control and move in a task-specific context to score in the games. Numerous repetitions and feedback *via* the game scores play an integral role in motor relearning and enhancing neuroplasticity which might have contributed to improving the balance (26).

In the Scoop'd group, the components of PBS such as standing with one foot in front, standing on one foot, and placing an alternate foot on the stool improved significantly. The movement of the participant in various directions to catch the ice-cream scoops in this game might have caused this improvement. Also, AR delivers visual and auditory feedback on balance parameters which can provide higher specificity and continuity and lower delay than the feedback delivered by a therapist (25). This might also explain the improved PBS scores in this group.

In the Bubble Pop group, standing with one foot in front, placing an alternate foot on the stool, and reaching forward with an outstretched arm significantly improved post-intervention. Just like Scoop'd, this game also involved the participant in multi-directional movements that might be the reason for this improvement. The participant also had to extend their UE to pop the bubbles which may have contributed to improving the forward-reaching score in PBS.

Standing with one foot in front also improved significantly in the Balance It group, which just like the previous two games involved multi-directional motion. According to a systematic review, balance training with feedback can result in improved ability to shift weight, reduced postural sway, and reduced attentional demands while standing (8). Similarly, AR games might have also improved these factors resulting in an improvement in the PBS scores.

A significant improvement with a large effect size has been determined in the DASH score in all the groups post-intervention. The possible reasons for this improvement might be AR-based cognitive engagement during play, enhanced problem-solving, neuroplasticity changes, and increased motivation. AR creates repetitive task-specific and task-oriented practices in a real environment, the flexibility of adjusting task difficulties, and the potential for interaction and social play (27). AR also offers social support from peers, parents, and therapists. All these elements from AR may have helped to change the personal and environmental barriers that a child with CP faces. This, in turn, might have gradually improved their UE function and decreased activity limitations, and in the future, it might also gradually lead to improved participation in school, society, and communities (27). Previously, studies have demonstrated the advantages of AR for rehabilitation purposes including its effectiveness for improving range of motion and muscle strength of upper extremity (28), however, there is a lack of evidence on its usefulness for upper extremity function.

The comparison between the groups showed that there was no significant difference in the total DASH score and in the domains of PBS except in standing with one foot in front and the total PBS score. There was a significant difference determined in standing with one foot in front between Bubble Pop and Balance It groups. The total score of PBS also showed a significant difference between Bubble Pop and Balance It groups. Furthermore, the Balance It game showed a better improvement in balance as compared to the other games. Though all the games involved motion in different directions, however, the time spent in a specific posture and movement pattern differed greatly in each game and this might would have caused such differences among the groups.

AR allows optimum exercise orientation toward objectives with high patient motivation and is also enjoyable to use (29). AR technologies also provide new experiences to patients during physiotherapy sessions, increasing participation and improving physical outcomes, and can create engaging opportunities to provide low-cost physiotherapy at home. Furthermore, the physiotherapist can perform and evaluate different outcomes by using the AR intervention through the data analysis (29).

Augmented reality is a promising intervention that could be used during rehabilitation for children with CP as an addition to other appropriate therapies (26). The results of this study suggest that AR intervention may assist children with CP in UE function, in improving their body balance, and thus may also lead to increased participation in family and community-based activities. All the children adhered to the therapy program which is highly encouraging for future clinical applications of AR as an augmentation to therapy. Though the participants were not systematically interviewed regarding their experiences using AR, all children reported that they enjoyed playing AR games and found it motivating. Future studies should collect systematic qualitative data to understand participant experiences in using AR games. No adverse effects of the interventions were noted, and the administration of AR intervention was easier for the physiotherapists as compared to the traditional methods.

## Limitations

The limitations of this study are that there was no control group and the total levels of the game played by each participant were not noted. These should be incorporated into future research along with including larger sample size and longer study duration to determine the comparative effectiveness of AR games and standard physiotherapy, and to attain further improvements. It is also suggested that these AR games may be improved by adding elements of variation and challenge to achieve greater and generalized improvement in the function of UE and body balance in children with SHCP.

## Conclusion

Augmented reality games used in this study were found to be effective in improving the function of the upper extremity and balance of children with SHCP. The Balance It game showed more promising results in improving the balance as compared to the other games, however, no significant difference was determined between the three AR games in terms of the UE function of the participants.

## Data Availability Statement

The raw data supporting the conclusions of this article will be made available by the authors, without undue reservation.

## Ethics Statement

The studies involving human participants were reviewed and approved by Institutional Review Board and Ethics Committee of NIRM. Written informed consent to participate in this study was provided by the participants' legal guardian/next of kin. Written informed consent was obtained from the minor(s)' legal guardian/next of kin for the publication of any potentially identifiable images or data included in this article.

## Author Contributions

WM and MA: substantial contributions to the conception and design of the study. WM, RB, and QM: acquisition of data for the study. WM and WA: interpretation of data for the study. WA: analysis of the data for the study. WM and RB: drafted the work. WM, WA, MA, and QM: revised it critically for important intellectual content. WM, RB, WA, MA, and QM: final approval of the version to be published and agreement to be accountable for all aspects of the work in ensuring that questions related to the accuracy or integrity of any part of the work are appropriately investigated and resolved. All authors contributed to the article and approved the submitted version.

## Conflict of Interest

The authors declare that the research was conducted in the absence of any commercial or financial relationships that could be construed as a potential conflict of interest.

## Publisher's Note

All claims expressed in this article are solely those of the authors and do not necessarily represent those of their affiliated organizations, or those of the publisher, the editors and the reviewers. Any product that may be evaluated in this article, or claim that may be made by its manufacturer, is not guaranteed or endorsed by the publisher.
